# Brain Injury Prediction: Assessing the Combined Probability of Concussion Using Linear and Rotational Head Acceleration

**DOI:** 10.1007/s10439-012-0731-0

**Published:** 2013-01-09

**Authors:** Steven Rowson, Stefan M. Duma

**Affiliations:** Center for Injury Biomechanics, Virginia Tech – Wake Forest, 440 ICTAS Building, Stanger St., Blacksburg, VA 24061 USA

**Keywords:** Mild traumatic brain injury, Biomechanics, Football, Helmet, Angular, Risk curve

## Abstract

Recent research has suggested possible long term effects due to repetitive concussions, highlighting the importance of developing methods to accurately quantify concussion risk. This study introduces a new injury metric, the combined probability of concussion, which computes the overall risk of concussion based on the peak linear and rotational accelerations experienced by the head during impact. The combined probability of concussion is unique in that it determines the likelihood of sustaining a concussion for a given impact, regardless of whether the injury would be reported or not. The risk curve was derived from data collected from instrumented football players (63,011 impacts including 37 concussions), which was adjusted to account for the underreporting of concussion. The predictive capability of this new metric is compared to that of single biomechanical parameters. The capabilities of these parameters to accurately predict concussion incidence were evaluated using two separate datasets: the Head Impact Telemetry System (HITS) data and National Football League (NFL) data collected from impact reconstructions using dummies (58 impacts including 25 concussions). Receiver operating characteristic curves were generated, and all parameters were significantly better at predicting injury than random guessing. The combined probability of concussion had the greatest area under the curve for all datasets. In the HITS dataset, the combined probability of concussion and linear acceleration were significantly better predictors of concussion than rotational acceleration alone, but not different from each other. In the NFL dataset, there were no significant differences between parameters. The combined probability of concussion is a valuable method to assess concussion risk in a laboratory setting for evaluating product safety.

## Introduction

With as many as 3.8 million sports-related concussions occurring annually in the United States and research suggesting possible long term neurodegenerative processes resulting from repetitive concussions, reducing the incidence of concussion in sports has become a public health priority.^32^,^42^,^43^ While limiting the number of head impacts in sports through rule changes and improved education for coaches and players have an important role in reducing concussion incidence, incidental head impacts cannot be removed from sports.^10^ It has been suggested that the monitoring of head impacts to identify high risk events and alert medical personnel to perform a concussion evaluation may reduce the incidence and severity of concussions by preventing subsequent impacts that may cause brain injury due to impact.^21^ Part of the remaining burden of reducing concussion incidence relies on the improvement of head protection. Helmets currently used in sports are designed to pass test standards that evaluate a helmet’s ability to prevent skull fracture.^40^,^41^ As a result, skull fractures have essentially been eliminated in helmeted sports, but these helmets are not designed to guard against concussion.^51^ One of the challenges in designing helmets to account for concussive forces is accurately modeling concussion risk in the laboratory. This article focuses on the kinematic parameters used to predict brain injury.

Kinematic parameters of the head are commonly used to assess brain injury risk because they are thought to be indicative of the inertial response of the brain.^53^ Traditionally, research investigating the biomechanics associated with brain injury has focused on two injury modes: injury resulting from linear acceleration and injury resulting from rotational acceleration. Linear acceleration-based brain injury is thought to result from a transient intracranial pressure gradient, while rotational acceleration-based brain injury is thought to result from a strain response.^30^,^57^ Historically, these two injury modes have been investigated independently of one another. Most notably, the Wayne State Tolerance Curve (WSTC) was developed from a series of tests on cadavers and dogs relating linear acceleration to injury tolerance.^23^ Kinematic based injury metrics were subsequently developed from analyses of the WSTC.^18^,^58^ While these injury metrics were primarily developed to correlate with skull fracture, they were also thought to correlate with severe brain injury. These works are the basis of all head injury safety standards for automobiles and helmets in the United States. There currently is no federal or industry head injury safety standard that considers rotational acceleration, even though there is strong evidence linking it to injury. Studies have since investigated the relationship between rotational acceleration and brain injury tolerance by exposing animals (primate or rat) to sudden rotation.^13^,^19^,^20^,^33^,^34^,^44^–^46^ However, there are challenges in relating data from an animal model to that of a human.^47^ Furthermore, these studies investigated linear and rotational acceleration separately, even though real-world head impacts consist of both linear and rotational acceleration components.^50^

Noting the high incidence of concussion in football,^29^ researchers have investigated these head impacts to relate head kinematics to injury. Pellman *et al*.^48^ analyzed video of concussive impacts in the National Football League (NFL) and reconstructed these impacts using crash test dummies. From this work, they developed separate concussion risk curves for linear and rotational acceleration. While the work was of high quality, the tedious testing methodology was impractical for assessing exposure to head impact by football players. As a result, the study did a good job of characterizing concussive biomechanics, but the risk curves likely overestimate risk because the study did not account for all the head impacts that to do not result in concussion.^17^,^52^ Shortly thereafter, other researchers went on to instrument the helmets of football players with commercially available accelerometer arrays (HIT System, Simbex, Lebanon, NH) to measure the head accelerations associated with every head impact that instrumented players experienced.^6^,^11^,^12^,^15^,^16^,^24^,^36^,^49^,^50^ From this work, concussion risk curves were developed for linear acceleration and rotational acceleration.^51^,^53^ These studies have provided valuable sub-concussive and concussive data measured directly from humans in real-world head impacts. This technology has since been adapted to other sports.^1^,^4^,^27^ While these real-world head impacts consist of linear and rotational components, linear and rotational acceleration have typically been analyzed independently of one another.

It has been suggested that linear and rotational injury metrics be used in combination.^44^ Along these same lines, other researchers have attempted to create composite injury prediction metrics that consider both linear and rotational acceleration. Newman *et al*.^39^ proposed the head impact power that considers linear acceleration, rotational acceleration, impact duration, and inertial measurements. This injury metric was developed from dummy data generated through reconstructions of NFL impacts.^37^,^38^ Greenwald *et al*.^22^ also developed a composite injury metric, weighted principal component score (wPCS), based on studies measuring head impacts in instrumented football players. wPCS is derived from a principal components analysis that considers peak linear acceleration, rotational acceleration, Gadd severity index (GSI),^18^ head injury criterion (HIC), and impact location. This study introduces a new injury metric derived from a multivariate logistic regression analysis that accounts for the underreporting of concussion and considers both linear and rotational head acceleration when assessing concussion risk. The predictive capability of this new metric is compared to that of single biomechanical parameters.

## Methods

### Combined Probability Risk Function

This study introduces a new injury metric, the combined probably of concussion, which considers the overall risk of concussion associated with the peak linear and rotational head accelerations resulting from impact, while accounting for underreporting rates of concussion and the dependent nature of linear and rotational acceleration. To do this, a dataset of previously published HITS data was compiled, which consisted of peak linear and rotational head accelerations for 62,974 sub-concussive impacts and 37 diagnosed concussive impacts measured by instrumented helmets during play in football.^5^,^24^,^51^,^53^

A multivariate logistic regression analysis was used to develop an injury risk curve that receives peak linear and rotational head acceleration experienced during an impact as input, and determines the probability of sustaining a concussion from that impact as output. In developing this type of risk curve, it is important that the weighting between the sub-concussive and concussive data distributions be representative of concussion incidence rates experienced in football. Furthermore, the rate at which concussions go undiagnosed must be considered, as concussions are thought to be widely underreported.^31^,^35^,^59^ To address this, concussion rates inclusive of diagnosed and undiagnosed concussions were estimated from the literature.

From surveying certified athletic trainers, Guskiewicz *et al*.^25^ reported that 5% of college and high school football players sustain concussions. In efforts to address the underreporting of concussion, McCrea *et al*.^35^ anonymously surveyed players on their concussion history and reported that 15% of high school football players experience concussions. When Langburt *et al*.^31^ surveyed players with descriptions of symptoms, but omitted the word “concussion” from the survey, it was reported that 47% of high school players sustain concussions. To side with conservatism, an underreporting rate of 10× was used in this analysis. An incidence rate of 5.56 concussions per 1000 games played was also used, where a game played is defined as one athlete participating in at least one play of one game.^3^ To relate this concussion incidence rate to the number of head impacts that would be experienced in 1000 games, a rate of 14.3 impacts per player per game was used,^9^ resulting in a concussion incidence rate of 3.88 concussions per 10,000 head impacts. When accounting for underreporting, the concussion incidence rate increases to 38.8 concussions per 10,000 head impacts.

Based on the estimated concussion incidence rate, the 63,011 head impacts in the HITS dataset should include 244 concussions, and therefore, the HITS dataset was adjusted to include 207 additional concussions. A conservative approach was utilized to account for underreporting, in which the 207 sub-concussive impacts that had acceleration magnitudes of the greatest rank were transformed to concussive data points. The following procedure was used: (1) Concussive HITS data were fit to a bivariate distribution consisting of peak linear and rotational accelerations using a Gaussian copula, (2) The 207 sub-concussive impacts of greatest rank were reassigned as concussive impacts and generated in the copula, and (3) Copula data were then transformed back to an acceleration scale using previously published concussive distributions for linear and rotational acceleration.^51^,^53^

A concussion risk function was developed from the adjusted HITS dataset using a multivariate logistic regression analysis. Equation (1) displays the risk function; where *β*_0_, *β*_1_, and *β*_2_ are regression coefficients, *a* is peak linear acceleration, *α* is peak rotational acceleration, and *CP* is the combined probability of concussion. The regression coefficients were determined using a generalized linear model technique.

1 $$ CP = \frac{1}{{1 + e^{{ - \left( {\beta_{0} + \beta_{1} a + \beta_{2} \alpha + \beta_{3} a\alpha } \right)}} }} $$

### Predictive Capability Assessment

**Figure 1:**
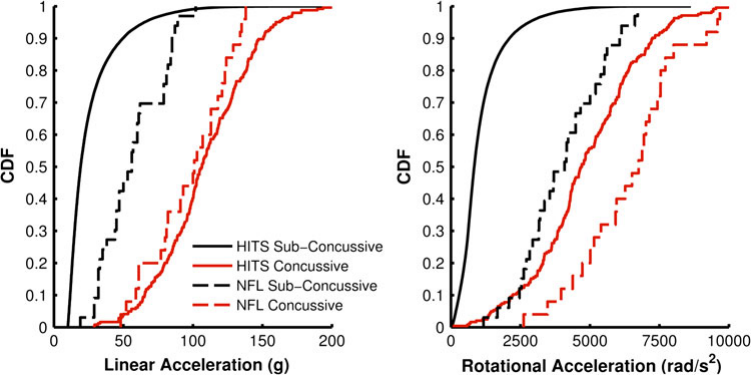
Comparison of the empirical CDF between the HITS and NFL datasets for peak linear acceleration and peak rotational acceleration

The predictive capability of the combined probability of concussion was compared to that of linear acceleration and rotational acceleration individually. The capabilities of these parameters to accurately predict concussion incidence were investigated using two head impact datasets: HITS data and NFL data.^48^ As described above, the adjusted HITS dataset consists of 63,011 impacts, including 244 concussions, collected through the instrumentation of football helmets. Concussive impacts in the HITS dataset had average accelerations of 104 ± 30 g and 4726 ± 1931 rad/s^2^. Sub-concussive impacts in the HITS dataset had average accelerations of 26 ± 19 g and 1072 ± 850 rad/s^2^. The NFL dataset consists of 58 impacts, including 25 concussions, collected from impact reconstructions of NFL impacts using crash test dummies.^48^ Concussive impacts in the NFL dataset had average accelerations of 98 ± 28 g and 6432 ± 1813 rad/s^2^. Sub-concussive impacts in the NFL dataset had average accelerations of 57 ± 22 g and 4029 ± 1438 rad/s^2^. Figure 1 compares the empirical cumulative distribution functions (CDF) for each dataset.

Due to large number of impacts in the HITS dataset that did not result in concussion, subsets of the HITS dataset were also analyzed. Using peak resultant linear acceleration to gauge impact severity, the top 50% and top 25% of HITS impacts were investigated. The top 50% of sub-concussive impacts in the HITS dataset consisted of impacts with peak linear accelerations greater than 19 g and had average accelerations of 38 ± 20 g and 1528 ± 984 rad/s^2^. The top 25% of sub-concussive impacts in the HITS dataset consisted of impacts with peak linear accelerations greater than 31 g and had average accelerations of 52 ± 21 g and 2036 ± 1124 rad/s^2^.

The combined probability of concussion for each impact in each dataset was determined using Equation 2. For each dataset, the capabilities of linear acceleration, rotational acceleration, and the combined probability of concussion to accurately predict concussion incidence were quantified using receiver operating characteristic (ROC) curves. For each parameter, the area under its ROC curve (AUC) was compared to the predictive capability of random guessing (AUC = 0.5) using a significance level of *p* < 0.05. Furthermore, the predictive capability of each parameter was compared with Hanley’s method of direct comparison of AUCs using a significance level of *p* < 0.05 for each dataset.^26^

## Results

**Figure 2:**
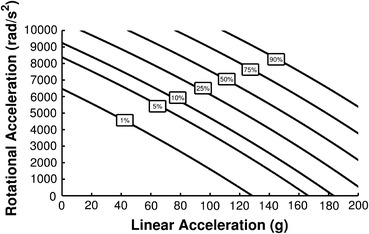
Combined probability of concussion contours relating overall concussion risk to linear and rotational head acceleration

The regression coefficients for the combined probability of concussion equation were determined (Eq. (2)), with *β*_0_ = −10.2, *β*_1_ = 0.0433, *β*_2_ = 0.000873, and *β*_3_ = −0.000000920. Risk contours relating peak linear and rotational head acceleration to concussion risk are shown in Fig. 2.

2 $$ CP = \frac{1}{{1 + e^{{ - \left( { - 10.2 + 0.0433 \cdot a + 0.000873 \cdot \alpha - 0.000000920 \cdot a\alpha } \right)}} }} $$

**Figure 3:**
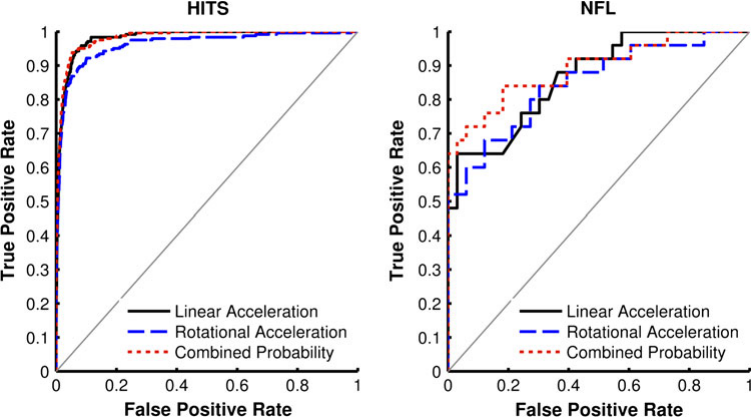
ROC curves for the HITS (left) and NFL (right) datasets for linear acceleration, rotational acceleration, and the combined probability of concussion

For the HITS and NFL datasets, ROC curves were generated for linear acceleration, rotational acceleration, and the combined probability of concussion (Fig. 3). Table 1 displays the AUC with 95th percentile confidence intervals for each parameter for both datasets. All parameters were better predictors of concussion than random guessing for the HITS and NFL datasets (*p* < 0.0001). For the HITS dataset, the AUCs associated with the combined probability of concussion and linear acceleration were significantly different than the AUC associated with rotational acceleration (*p* < 0.015). The AUC associated with the combined probability of concussion was not significantly different than the AUC associated with linear acceleration (*p* = 0.88). For the NFL dataset, linear acceleration, rotational acceleration, and the combined probability of concussion were not significantly different from each other (*p* > 0.20).

**Table 1:** For the HITS and NFL datasets, the area under each ROC curve (AUC) for linear acceleration, rotational acceleration, and the combined probability of concussion was significantly different (denoted by *p*) than the AUC associated with random guessing (AUC = 0.5)

	HITS		NFL	
	AUC [95% CI]	*p*	AUC [95% CI]	*p*
Linear acceleration	0.981 [0.969–0.993]	<0.0001*	0.867 [0.766–0.967]	<0.0001*
Rotational acceleration	0.962 [0.945–0.979]	<0.0001*	0.849 [0.742–0.955]	<0.0001*
Combined probability	0.982 [0.970–0.994]	<0.0001*	0.892 [0.801–0.983]	<0.0001*

95th percentile confidence intervals (95% CI) for each AUC are provided in brackets

*A significance threshold of *p* < 0.05 was used

**Figure 4:**
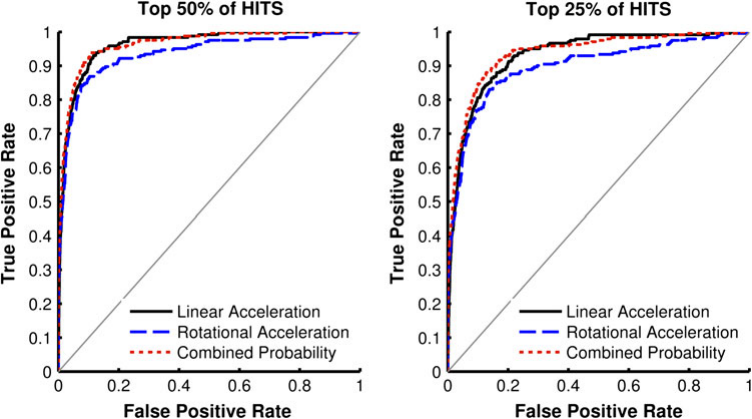
ROC curves for the top 50% of HITS data (left) and top 25% of HITS data (right) for linear acceleration, rotational acceleration, and the combined probability of concussion

The top 50% and top 25% of HITS data based on peak linear acceleration were analyzed separately and ROC curves were generated for linear acceleration, rotational acceleration, and the combined probability of concussion (Fig. 4). Table 2 displays the AUC with 95th percentile confidence intervals for each parameter for the top 50% and top 25% of HITS data. For both data subsets, all parameters were better predictors of concussion than random guessing (*p* < 0.0001). For the top 50% of HITS data, the AUCs associated with the combined probability of concussion and linear acceleration were significantly different than the AUC associated with rotational acceleration (*p* < 0.0076). The AUC associated with the combined probability of concussion was not significantly different than the AUC associated with linear acceleration (*p* = 0.81). For the top 25% of HITS data, the AUCs associated with the combined probability of concussion and linear acceleration were significantly different than the AUC associated with rotational acceleration (*p* < 0.011). The AUC associated with the combined probability of concussion was not significantly different than the AUC associated with linear acceleration (*p* = 0.58).

**Table 2:** For the top 50% and top 25% of HITS data, the area under each ROC curve (AUC) for linear acceleration, rotational acceleration, and the combined probability of concussion was significantly different (denoted by *p*) than the AUC associated with random guessing (AUC = 0.5)

	Top 50% of HITS		Top 25% of HITS	
	AUC [95% CI]	*p*	AUC [95% CI]	*p*
Linear acceleration	0.962 [0.945–0.979]	<0.0001*	0.932 [0.909–0.954]	<0.0001*
Rotational acceleration	0.934 [0.913–0.956]	<0.0001*	0.898 [0.871–0.924]	<0.0001*
Combined probability	0.964 [0.947–0.980]	<0.0001*	0.937 [0.916–0.958]	<0.0001*

95th percentile confidence intervals (95% CI) for each AUC are provided in brackets

*A significance threshold of *p* < 0.05 was used

For all comparisons, the combined probability of concussion was associated with the greatest AUC. However, linear acceleration was not a significantly worse predictor of concussion than the combined probability of concussion for all datasets. Rotational acceleration was associated with the smallest AUC for all datasets. Using rotational acceleration as a brain injury predictor resulted in the greatest false positive rate associated with high true positive rates, while using the combined probability of concussion produced lowest false positive rates in all HITS datasets (Table 3).

**Table 3:** Comparison of false positive rates for each parameter at 75 and 90% true positive rates in each dataset

	75% True positive rate			90% True positive rate		
	Linear accel. (%)	Rotational accel. (%)	Combined probability (%)	Linear accel. (%)	Rotational accel. (%)	Combined probability (%)
HITS	2.0	2.3	1.6	4.9	8.8	4.0
NFL	24.4	27.3	12.1	42.4	51.5	39.4
50th % HITS	4.0	4.5	3.1	9.9	17.6	8.0
25th % HITS	7.8	9.1	6.2	19.7	29.6	15.9

## Discussion

This study introduces a new injury metric, the combined probability of concussion, which computes the overall risk of concussion based on the peak linear and rotational accelerations experienced by the head during impact. The combined probability of concussion is unique in that it determines the likelihood of sustaining a concussion for a given impact, regardless of whether the athlete would report the injury or not. This was accomplished by adjusting the HITS dataset to account for an estimated underreporting rate during development of the risk curve. To side with conservatism, a greater underreporting rate was used in this analysis than previous independent linear and rotational acceleration risk curves that considered underreporting.^51^,^53^ Linear and rotational acceleration are considered because they both likely contribute to concussion risk and are thought to be associated with different injury mechanisms.^30^,^44^,^57^ Linear acceleration of the head is associated with a transient intracranial pressure gradient, while rotational acceleration of the head is associated with a strain response. Experiments designed to induce brain injury in animals have produced injury through isolated linear acceleration and isolated rotational acceleration events. Furthermore, Hardy *et al*.^28^ measured the pressure and strain response of the human cadaver head to impact. Impacts similar in severity to those experienced in football were modeled, and kinematic parameters were related to the pressure and strain response of the brain. Peak pressure increased with increasing linear acceleration of the head. Peak strains were less than 9% and brain motion correlated with rotational speed. For these reasons, both linear and rotational acceleration are considered in the combined probability of concussion.

Data from two different methodologies used to investigate the biomechanics of concussions were analyzed in this study. The HITS dataset was comprised of data collected from instrumented football players, while the NFL dataset was generated through laboratory reconstructions using crash test dummies. Even though data were generated from two different methodologies, the peak linear and rotational accelerations associated with concussion are similar. The primary difference between the two datasets is the sub-concussive subset. The HIT System allowed for the recording of every head impact a player experienced during games and practices while he was instrumented. As a result, the HITS dataset includes a vast number of impacts that did not result in concussion, and is more representative of the total head impact exposure that football players experience.^17^,^53^ The NFL dataset was generated from laboratory reconstructions that made it impractical to consider the thousands of head impacts experienced by NFL players, and instead only modeled some of the more severe impacts that could be characterized from video analysis.^48^ Both datasets are valuable tools for evaluating injury predictors, but it is important to understand these differences between the HITS and NFL data.

Concussive impacts are well-characterized by peak biomechanical measures, and as acceleration magnitude increases, injury risk also increases.^2^,^14^,^51^,^53^ For this reason, all predictors were very sensitive to identifying concussive impacts within datasets. However, because the datasets had varying distributions of sub-concussive impacts, the specificity of the predictors varied greatly (Table 3). For true positive rates of 75 and 90%, the small number of low magnitude sub-concussive impacts in the NFL dataset resulted in much greater false positive rates than the HITS datasets. In contrast, the high number of low-magnitude sub-concussive impacts in the HITS dataset resulted in very low false positive rates. As the HITS dataset was parsed into the top 50% and top 25% of impacts, false positive rates increased. Given the vast number of impacts in the HITS datasets, the number of false positives was greater than the number of concussions. This lack of specificity can be partially attributed the underreporting of concussion. It is possible that some of the impacts labeled as sub-concussive impacts in the HITS dataset and the NFL dataset resulted in a concussion that was not reported, even after the HITS dataset was adjusted to account for underreporting. While concussive impacts could be characterized using these biomechanical measures, other factors such as impact location, impact duration, muscle factors, and genetic predispositions likely affect concussion risk.^22^,^53^

For all datasets, the combined probability of concussion produced the greatest AUC, suggesting it was the best predictor of concussion of the parameters investigated. However, linear acceleration was not significantly different than the combined probability of concussion, suggesting it can predict concussion as well as the combined probability of concussion in the datasets analyzed in this study. With the exception of the NFL data, where there were no differences among parameters, rotational acceleration was a significantly worse predictor of concussion than the combined probability of concussion and linear acceleration. This is due to most head impacts in football being inherently similar to one another, in that they are linearly driven. Rotational acceleration of the head is a function of the linear acceleration and direction of force acting on the head. The relationships between linear acceleration and rotational acceleration are similar for impacts to the front, side, and back of the helmet.^53^ However, impacts to the top of the helmet result in a much different relationship, where for a given linear acceleration, rotational accelerations are much lower.^53^ In these datasets, concussive impacts to the top of the helmet resulted in high linear accelerations and relatively low rotational accelerations that reduced the predictive capability of peak rotational acceleration. The combined probability of concussion method accounted for this because it considered both linear and rotational accelerations for each impact.

The acceleration response of real-world head impacts consists of linear and rotational acceleration components. Depending on the impact location and direction of force, the respective contribution of linear and rotational acceleration will vary. For head impacts in football, the profile and duration of the head acceleration response are similar, and rotational acceleration is correlated to linear acceleration.^53^ As new helmet designs begin to incorporate mechanisms to manage rotational acceleration independently of linear acceleration, this relationship will likely vary. The combined probability of concussion will be a useful tool in evaluating such designs. While only football head impact datasets were analyzed in this study, the combined probability of concussion has applications beyond football, considering impacts are similar in impact duration. The combined probability of concussion could be a valuable method to assess brain injury risk in a laboratory setting for evaluating product safety, including head protection and automobile restraint design, considering that the impact characteristics are similar to those analyzed here. With an improved ability to assess concussion risk, engineering analyses can be used to evaluate and influence product design to reduce injury incidence.^54^–^56^

This study has several limitations. First, ROC analysis is dependent on the dataset that is being characterized. A specific type of impact mode (football helmet impacts) was analyzed in this study using 2 different datasets. Neither dataset included impacts that were predominantly comprised of rotational acceleration, as this impact mode is very rare in football. Further work is needed to assess to the predictive capability of the combined probability of concussion for impacts that are predominantly comprised of rotational acceleration. Second, the underreporting of concussion may have affected the ROC analysis, even though the HITS dataset was adjusted to account for underreporting rates. Unreported concussions in the HITS and NFL datasets would result in conservative estimates of specificity, where the true value of the false positive rates would be lower. While these datasets may be limited by the presence of unreported concussions, these are currently the best datasets that are available for analyzing the biomechanics of concussion in humans. Third, the combined probability of concussion only considers peak linear and rotational acceleration. While risk curves are commonly used to relate mechanical stimuli to injury, other factors can affect injury risk.^7^,^8^ Impact location, impact duration, muscle factors, and genetic predispositions likely affect concussion risk. When using the combined probability of concussion to evaluate injury risk, one should be aware of impact duration and these other factors that may affect risk. With that said, peak linear and rotational acceleration characterize concussion well and are good predictors of injury.

## Conclusion

This study introduces a new method of assessing the overall risk of concussion from peak linear and rotational acceleration for a given impact. The combined probability of concussion is unique in that it determines the likelihood of sustaining a concussion for a given impact, regardless of whether the injury would be reported or not. Two separate datasets were used to assess the predictive capability of linear acceleration, rotational acceleration, and the combined probability of concussion method. ROC analyses suggested all parameters were good predictors of injury for the datasets analyzed, with the combined probability of concussion and linear acceleration being significantly better predictors than rotational acceleration. Of the parameters analyzed, the combined probability of concussion method produced the greatest AUC because it can account for more impact scenarios than linear or rotational acceleration alone. Future applications include assessing concussion risk in a laboratory setting for evaluating product safety, including head protection and automobile restraint design.
